# Are Appropriate Use Recommendations useful in clinical practice?

**DOI:** 10.1016/j.tjpad.2025.100165

**Published:** 2025-04-15

**Authors:** Serge Gauthier

**Affiliations:** Emeritus Professor in Neurology and in Psychiatry, McGill University, Montreal Canada

The Alzheimer's disease (AD) therapeutic field is fortunate to have dedicated clinicians writing Appropriate Use Recommendations (AUR) for the new anti-amyloid therapies approved first in the USA and now in some other countries [[1], [2], [3], [4]]. This first generation of drugs modifying disease progression through amyloid clearing using monoclonal antibodies is complex in its use because of the need for drug delivery at bimonthly or monthly intervals using intravenous infusions and the risk of Amyloid-Related Imaging Abnormalities (ARIA).

These AUR are well cited in the medical literature (375 for [1], 172 for [2] and 446 for [3] as of March 29th 2025). They are also downloaded in large numbers (40K for [1], 14K for [2], 83K for [3] as of March 31st 2025). They are an editor's dream because of the quality of the writing, the willingness of outside reviewers to read and comment them, and the quick turn around for answers from the authors.

More importantly they answer the needs from clinicians around the world who are eager to know about these new drugs and see if they can be useful to some of their patients. Relatively few clinicians will have the human and technical resources to use them, but local, regional and national care structures are being progressively set up [5]. AUR written from other countries perspectives are also emerging [6], and more to come from countries such as China, Japan and the UK, where both lecanemab and donanemab have been approved by their regulatory agencies.

Whait is special about the current AUR for donanemab? They summarize the mechanism of action of the drug and the study trial designs that were used to demonstrate safety and efficacy. The reader will have a clear vision who the drug is for (sporadic AD at the mild cognitive impairment or mild dementia stage, with amyloid positivity using PET or CSF, without overt vascular pathology particularly MRI evidence for cerebral amyloid angiopathy-related inflammation). Emphasis is on safe use with the option of a different titration schedule in persons with higher risks of ARIA such as APOE4 homozygotes. Anticoagulants are excluded because of risk of macrohemorrhages, at least until more safety data emerge from additional clinical trials and longitudinal registries following treated patients. Clinicians interested in stroke will find a section dedicated on why thrombolytics should be avoided, and how to distinguish ARIA from stroke. New features include the option of stopping treatment after normalization of an amyloid PET after 12–18 months, and for the first time switching between Aβ-targeting monoclonal antibodies. Finally, there are practical tips on resources required for the safe and effective use of donanemab.

In conclusion, clinicians who plan to use donanemab will find in these AUR very useful advice on how to go about it, particularly in terms of patient selection and safety monitoring. Clinicians who do not plan to us this drug will gain knowledge about the drug and be able to discuss with their potentially eligible patients the option of a referral to a local or regional specialty site.

Are AUR useful when new drugs are approved, particularly in a new drug class and a new indication (disease modification rather than symptomatic benefit)? Clearly yes from our experience with anti-amyloid monoclonal antibodies. Should AUR be written for each of the new drugs after regulatory approval? I would argue yes, and I can't wait for the AUR of the first anti-tau drug, and then the AUR about combinations of anti-amyloid and anti-tau therapies!

## Declaration of competing interest

Serge Gauthier reports a relationship with Alzheon that includes: consulting or advisory. Serge Gauthier reports a relationship with AmyriAD that includes: consulting or advisory. Serge Gauthier reports a relationship with Eisai Canada that includes: consulting or advisory. Serge Gauthier reports a relationship with ENIGMA USA that includes: consulting or advisory and travel reimbursement. Serge Gauthier reports a relationship with Lilly Canada that includes: consulting or advisory. Serge Gauthier reports a relationship with Otsuka Canada that includes: consulting or advisory. Serge Gauthier reports a relationship with Novo Nordisk Canada that includes: consulting or advisory. Serge Gauthier reports a relationship with TauRx Therapeutics Ltd that includes: consulting or advisory and travel reimbursement. Serge Gauthier reports a relationship with ADvantage that includes: consulting or advisory. Serge Gauthier reports a relationship with AbbVie that includes: consulting or advisory. Editor in chief, JPAD.
